# Upregulated Expression of Transient Receptor Potential Cation Channel Subfamily V Receptors in Mucosae of Patients with Oral Squamous Cell Carcinoma and Patients with a History of Alcohol Consumption or Smoking

**DOI:** 10.1371/journal.pone.0169723

**Published:** 2017-01-12

**Authors:** Akiko Sakakibara, Shunsuke Sakakibara, Junya Kusumoto, Daisuke Takeda, Takumi Hasegawa, Masaya Akashi, Tsutomu Minamikawa, Kazunobu Hashikawa, Hiroto Terashi, Takahide Komori

**Affiliations:** 1 Department of Oral and Maxillofacial Surgery, Kobe University Graduate School of Medicine, Kobe, Japan; 2 Department of Plastic Surgery, Kobe University Graduate School of Medicine, Kobe, Japan; Fu Jen Catholic University, TAIWAN

## Abstract

**Objectives:**

Transient receptor potential cation channel (subfamily V, members 1–4) (TRPV1–4) are expressed in skin and neurons and activated by external stimuli in normal mucosae of all oral cavity sites. The oral cavity is exposed to various stimuli, including temperature, mechanical stimuli, chemical substances, and changes in pH, and, notably, the risk factors for oncogenic transformation in oral squamous epithelium are the same as the external stimuli received by TRPV1–4 receptors. Hence, we examined the relationship between oral squamous cell carcinoma (SCC) and TRPV1–4 expression.

**Materials and Methods:**

Oral SCC patients (n = 37) who underwent surgical resection were included in this study. We investigated the expression of TRPV1–4 by immunohistochemical staining and quantification of *TRPV1–4* mRNA in human oral mucosa. In addition, we compared the TRPV1–4 levels in mucosa from patients with SCC to those in normal oral mucosa.

**Results:**

The receptors were expressed in oral mucosa at all sites (tongue, buccal mucosa, gingiva, and oral floor) and the expression was stronger in epithelia from patients with SCC than in normal epithelia. Furthermore, alcohol consumption and tobacco use were strongly associated with the occurrence of oral cancer and were found to have a remarkable influence on TRPV1–4 receptor expression in normal oral mucosa. In particular, patients with a history of alcohol consumption demonstrated significantly higher expression levels.

**Conclusion:**

Various external stimuli may influence the behavior of cancer cells. Overexpression of TRPV1-4 is likely to be a factor in enhanced sensitivity to external stimuli. These findings could contribute to the establishment of novel strategies for cancer therapy or prevention.

## Introduction

The human body receives a variety of external stimuli, such as temperature, mechanical stimuli, chemical substances, and changes in pH. These external stimuli are converted via sensory receptor neurons into electrical signals, which transmit information to the central nervous system. Following the discovery that the conversion of temperature stimuli to neuronal signals is mediated by transient receptor potential cation channel (subfamily V, member 1) (TRPV1) [1], various TRP channel families have been identified. Proteins belonging to the TRP families differ in their sensitivity to external stimuli, such as temperature, mechanical stimuli, and some chemical substances; they function to convert external stimuli to intracellular and extracellular signals, depending on the type and intensity of the stimulus. In particular, TRPV1–4 receptors respond to heat, with activation thresholds between 25°C and 52°C [2, 3]. TRPV1 activation can occur because of stimulation with capsaicin (from chili peppers) as well as other chemical compounds.

In the past, TRP family members were thought to act only as sense receptors in sensory neurons; however, recent studies have found that TRP protein expression also occurs in non-neural tissue [4–6]. Specifically, TRPV family receptors are expressed in the skin (particularly the epidermis). Since the skin forms a boundary between the body and the outside world, it is intuitive that the skin would serve as a stimulus receptor; however, skin cells themselves do not transmit signals to other cells in the same way as neurons, and the function of TRPV receptors in the skin is not well understood.

TRPV receptor expression has been investigated in various cancers, and their involvement in tumor control is predicted [7–10]. However, organs that are commonly affected by cancer (e.g., colon, lung, and bladder) and are known to express TRPV receptors are not usually exposed to the types of stimuli that are associated with the function of TRPV channels. Therefore, the biological and oncological significance of TRPV receptor expression in such organs is unclear.

The oral mucosa, which is an epithelial tissue like the skin, is exposed to various stimuli, including hot, cold, sour, and spicy food and drinks, cigarettes, and alcohol. Risk factors for oral cancer include smoking, alcohol consumption, chronic mechanical irritation, chemical irritation from food, oral mucosal damage due to inflammation, and viral infection [11–14]. Hence, the risk factors for oncogenic transformation in oral squamous epithelium correspond to the external stimuli received by TRPV receptors. The oral cavity receives the stimuli recognized by TRPV1–4 receptors. Thus, determination of the expression pattern of TRPV receptors in oral cancer has potential to provide an important model for understanding the relationship between carcinogenesis and TRPV expression. Furthermore, TRPV-mediated intracellular signaling can lead both to cell apoptosis and proliferation [10, 15–18].

Expression patterns of TRPV1, 3, and 4 have previously been reported in normal tongue, buccal mucosa, and palate tissue of rats [19], while TRPV3 is reported as involved in healing mucosal injury in rat palatal mucosa [20]. In humans, quantitative expression analysis of TRPV1 has been reported in the lingual mucosa and oral squamous cell carcinoma (SCC) [16, 21]. Non-quantitative expression analysis of TRPV1–4 was also reported for normal human gingival mucosa [22]; however, there has been no comprehensive, quantitative examination of expression of TRPV1–4 in human oral mucosae. Moreover, it is unknown whether TRPV expression in oral mucosae from patients with SCC exacerbates or inhibits tumor proliferation in response to external stimuli. Therefore, we examined TRPV expression in healthy human oral mucosae and compared it to that in tumor specimens harvested from oral SCC patients. We also examined whether the level of expression differs between normal oral mucosae and oral mucosae from patients with SCC, whether the level of expression differs based on the degree of differentiation of the carcinoma, and whether alcohol consumption and smoking affect receptor expression in normal mucosae.

## Materials and Methods

### Tissue (Oral epithelia) collection

Oral SCC patients (n = 37) who underwent surgical resection at the Department of Oral and Maxillofacial Surgery, Kobe University Graduate School of Medicine between June 2012 and January 2015 were included in this study. Clinical data, including age, sex, tumor site, and pathological findings, were collected retrospectively. All patients involved in the present study underwent clinical treatment in accordance with consensus guidelines for head and neck carcinoma. All tumor specimens were examined by two experienced pathologists. As part of surgical resection, 5 mm^2^ specimens of both normal and tumor tissue were harvested from the tongue (n = 11), gingiva (n = 14), buccal mucosa (n = 6), and oral floor (n = 6) (all sites, n = 37); these specimens were immediately immersed in liquid nitrogen before cryopreservation at -80°C until use. All specimens were obtained in accordance with the World Medical Association Declaration of Helsinki Ethical Principles for Medical Research Involving Human Subjects. The study protocol was approved by Kobe University Graduate school of Medicine Ethics Committee (Permission No. 1303). We provided written information explaining this study to the patients who participated and written consent was obtained and recorded from all participants.

### Determination of mRNA expression levels by quantitative real-time PCR

Tissue specimens were immersed in 1 ml ISOGEN II (Nippon Gene, Chiyoda, Tokyo, Japan) while frozen and homogenized to extract total RNA, which was quantitated by spectrophotometry in preparation for use in quantitative polymerase chain reaction (qPCR) experiments.

For qPCR, we selected primers designed using the Perfect Real Time Primer Support System (Takara Bio, Shiga, Japan). The housekeeping gene β-actin (*ACTB*) was used as a standard for normalization of target gene levels.

Primers were as follows:

*ACTB*, forward (5′-TGG CAC CCA GCA CAA TGA A -3′) and reverse (5′-CTA AGT CAT AGT CCG CCT AGA AGC A -3′); *TRPV1*, forward (5′-TGT ACA TCC AAC CGT CAC TGT CC -3′) and reverse (5′-ATG TCC CAG TAG AGA CTG ACC ATC C -3′); *TRPV2*, forward (5′-TGA CAG CTG GAG CAT CTG GAA -3′) and reverse (5′-GCC AAC GGT CAG CAT CAC A -3′); *TRPV3*, forward (5′-TCC TGA TTC TTG GAC CAC AGA TG -3′) and reverse (5′-CAA TGT TGA CTG CCA AAT GAG ACA C -3′); and *TRPV4*, forward (5′-GGC TGG ATG AAT GCC CTT TAC -3′) and reverse (5′-GGT CTG GTC CTC ATT GCA CAC -3′). All primers were purchased from Takara Bio Inc. (Shiga, Japan).

Expression levels of *ACTB*, *TRPV1*, *TRPV2*, *TRPV3*, and *TRPV4* mRNA were analyzed by quantitative real-time PCR using a Thermal Cycler Dice^®^ Real Time System II (Takara Bio Inc., Shiga, Japan). A Prime Script 1 step Kit (Takara Bio Inc., Japan) was used for qPCR. Briefly, 0.4 μM forward and reverse primers, enzyme mix, 100 ng total RNA and One Step SYBER^®^ RT-PCR Buffer were added to a final reaction volume of 25 μl. Conditions for qPCR consisted of 40 cycles of denaturation at 95°C for 5 seconds, followed by annealing and extension at 60°C for 30 seconds. Four replicates of each experiment were performed and the mean values calculated from these used for comparative analysis.

### Immunohistochemical staining

Tissues were fixed in 10% buffered formalin. Paraffin embedded sections (4 μm thick) were prepared and fluorescence immunohistochemistry performed. Briefly, sections were incubated with anti-TRPV1 (rabbit polyclonal IgG; dilution 1:1000; Bioss, Boston, USA; bs-1931R), anti-TRPV2 (rabbit polyclonal IgG; dilution 1:1000; GeneTex, Irvine, USA; GTX101868), anti-TRPV3 (mouse monoclonal; dilution 1:500; Abcam, Cambridge, UK; ab85022), and anti-TRPV4 (rabbit polyclonal; dilution 1:1000; Bioss, Woburn, USA; bs-6425) for 7 days at 4°C in 0.1% Triton X-100/PBS. Antigen retrieval was only performed for TRPV3, using sodium citrate buffer (pH 6) at 90°C for 20 min. After the reaction with primary antibodies, sections were incubated with Alexa-488 conjugated secondary antibody (dilution 1:500; Invitrogen, Tokyo, Japan) for 2 h at room temperature, and counterstained with 0.1% Evan’s blue and DAPI. Microscopic analysis was performed with a fluorescent microscope (BZ-8100; Keyence, Osaka, Japan).

### Statistical analyses

Differences in relative levels of *TRPV1–4* mRNA expression in normal human mucosae were examined using a Friedman’s test followed by Scheffe’s correction for multiple comparisons. Differences in relative levels of expression between normal mucosae and oral mucosae from patients with SCC were examined using the Wilcoxon signed-rank test. Expression levels according to carcinoma pathological grade were analyzed using the Kruskal—Wallis test. Associations between alcohol consumption and smoking and relative expression levels of TRPV receptors were examined using the Mann—Whitney U test. The level of statistical significance was set at p < 0.05.

## Results

### Clinical and histological characteristics of patients

The clinical and pathological characteristics of the 37 patients included in this study are presented in Table 1. The 37 patients comprised 21 men and 16 women with a mean age of 71.6 years (range, 42–88 years). In all cases, the pathological diagnosis was SCC. The most common tumor site was the gingiva (14 cases), followed by the tongue (11 cases), the buccal mucosa (6 cases), and the oral floor (6 cases). The pathological TNM stages of the tumors were as follows: stage 1, 2 cases; stage 2, 11 cases; stage 3, 5 cases; and stage 4, 19 cases. The WHO classifications of the tumors were as follows: grade 1, 17 cases; grade 2, 15 cases; and grade 3, 5 cases. Twenty patients had a history of alcohol consumption, while 17 did not and 18 patients had a history of smoking, while 19 did not.

**Table 1:** Clinical, pathological, and epidemiologic characteristics of 37 patients with oral squamous cell carcinoma.

Characteristics		No. of patients (%)
Age		Range: 42–88 years
		Mean: 71.6 years
Sex	Male	21 (56.8%)
	Female	16 (43.2%)
Tumor site	Gingiva	14 (37.8%)
	Tongue	11 (29.7%)
	Buccal mucosa	6 (16.2%)
	Oral floor	6 (16.2%)
Pathological grade (WHO)	1	17 (45.9%)
	2	15 (40.5%)
	3	5 (13.5%)
Pathological TNM stage	I	2 (5.4%)
	II	11 (29.7%)
	III	5 (13.5%)
	IV	19 (51.4%)
Alcohol consumption	Yes	20 (54.1%)
	No	17 (45.9%)
Smoking	Yes	18 (48.6%)
	No	19 (51.4%)
**Total**		**37 (100%)**

### Immunohistological analysis

#### Expression of TRPV1–4 in human oral squamous cell carcinoma

**Fig 1:**
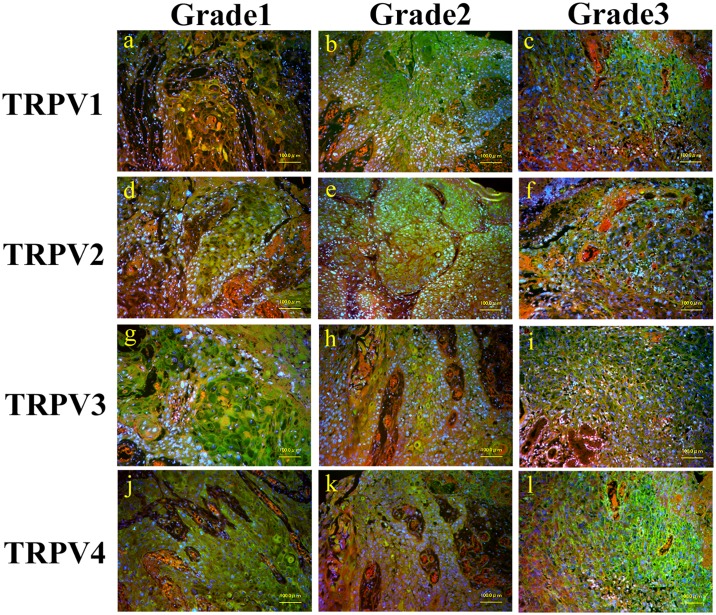
Immunostaining images of TRPV1–4 in human squamous cell carcinoma of the tongue (by grade). Merged image of anti-TRPV1–4 (green), counterstained with Evance blue (red) and DAPI (blue).

Using immunohistochemical staining to examine TRPV1–4 expression in oral mucosae from patients with SCC, we observed expression in all oral SCC sites, including the tongue (Fig 1), buccal mucosa, gingiva, and oral floor. As tumor tissues do not form layered structures, the observed expression patterns were diffuse. Furthermore, expression of TRPV1–4 was observed in all grades of SCC (Fig 1).

#### Expression of TRPV1–4 in human normal oral mucosa

**Fig 2:**
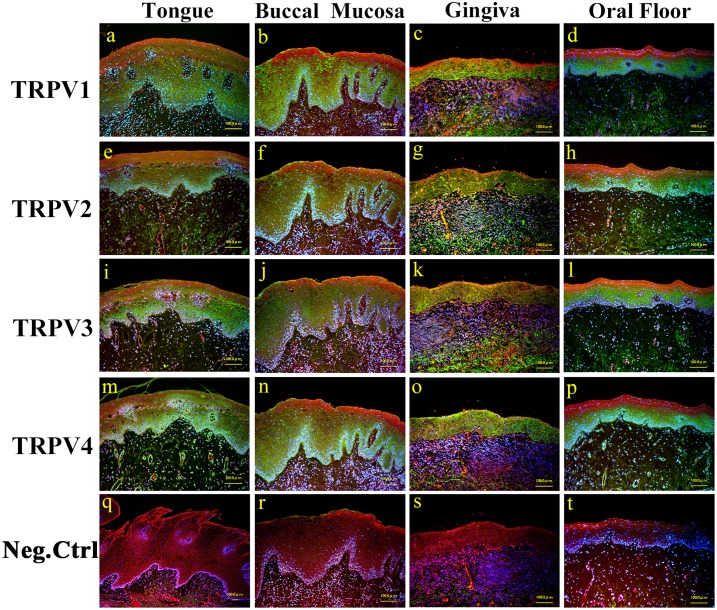
Immunostaining images of TRPV1–4 in normal human oral mucosae. Merged image of anti-TRPV1–4 (green), counterstained with Evance blue (red) and DAPI (blue).

We confirmed TRPV1–4 expression in normal mucosae of all oral cavity sites (tongue, buccal mucosa, gingiva, and oral floor) by immunohistochemical staining. Expression was observed throughout the mucosal epithelium in all sites (Fig 2). Although TRPV was most intense in the basal cell layer in the majority of sites, TRPV1 expression in the tongue (Fig 2a), and TRPV3 in the buccal mucosa (Fig 2j) and the oral floor (Fig 2l), were weak in the basal cell layer; in these cases, expression tended to be most intense in the prickle cell layer.

### Quantification of *TRPV1–4* mRNA in normal mucosa

**Fig 3:**
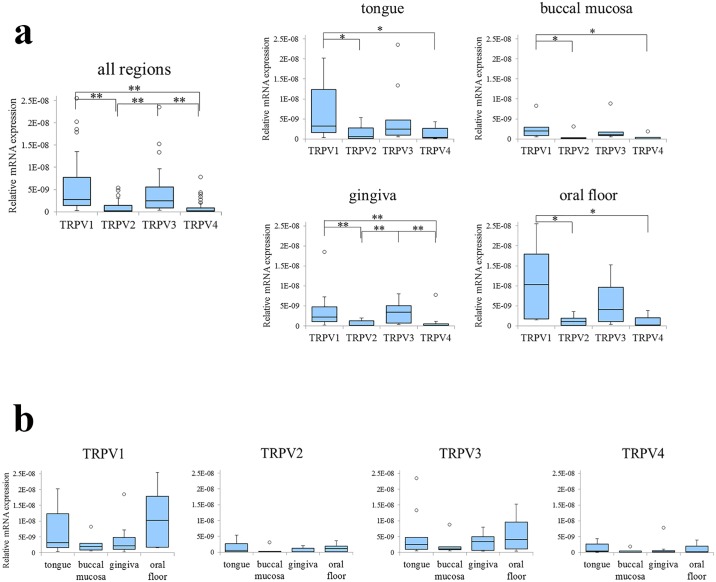
Box plot demonstrating *TRPV1–4* expression in normal human oral mucosae. The relative expression level of each target gene was normalized to *ACTB* levels in the same sample and expressed relative to control group expression (DDCT method; Applied Biosystems). Box plots illustrate the median, lower and upper quartile values of *TRPV1–4* expression. Whiskers were calculated using the Tukey method; filled circles represent outliers. (a) Relative levels of expression of *TRPV1*, *2*, *3*, and *4* in the oral cavity (all regions, tongue, buccal mucosa, gingiva, and oral floor). A comprehensive examination by qPCR of all regions in the oral cavity showed that expression of *TRPV1* and *TRPV3* was significantly higher than that of *TRPV2* and *TRPV4* (p < 0.01). In the tongue, buccal mucosa, and oral floor, *TRPV1* expression was significantly greater than that of *TRPV2* and *TRPV4* (p < 0.05). In the gingiva, relative expression levels of *TRPV1* and *TRPV3* were significantly higher than those of *TRPV2* and *TRPV4* (p < 0.01). (b) Relative levels of *TRPV1*, *2*, *3*, and *4* expression by site in normal human oral mucosae. No significant differences were observed in relative expression levels; however, expression levels were relatively high in the tongue and oral floor in comparison with the gingiva and buccal mucosa.

We were able to confirm and quantitate *TRPV1–4* mRNA expression in all oral cavity sites by qPCR (Fig 3a). Overall, relative mRNA expression was highest for *TRPV1* and *TRPV3*, while that of *TRPV2* and *TRPV4* was significantly lower. Comparison of expression levels of the four *TRPV* mRNAs in the tongue, buccal mucosa, gingiva, and oral floor revealed that *TRPV1* and *TRPV3* expression tended to be high, while that of *TRPV2* and *TRPV4* was relatively low. In all sites examined, expression of *TRPV1* was significantly higher than that of *TRPV2* and *4*; however, although expression of *TRPV3* was higher than that of *TRPV2* and *4*, the differences were only statistically significant for the gingiva and all oral cavity sites combined (Fig 3a)

Comparisons of expression of individual *TRPV* mRNAs in each oral cavity site revealed no significant differences in expression levels between sites. However, *TRPV1* and *TRPV3* expression tended to be highest in the oral floor, followed by the tongue, the buccal mucosa, and the gingiva, while *TRPV2* and *TRPV4* expression tended to be highest in the tongue, followed by the oral floor, the buccal mucosa, and the gingiva (Fig 3c).

### Quantification of *TRPV1–4* mRNA in oral SCC

#### Expression of TRPV1–4 in all oral SCC sites

**Fig 4:**
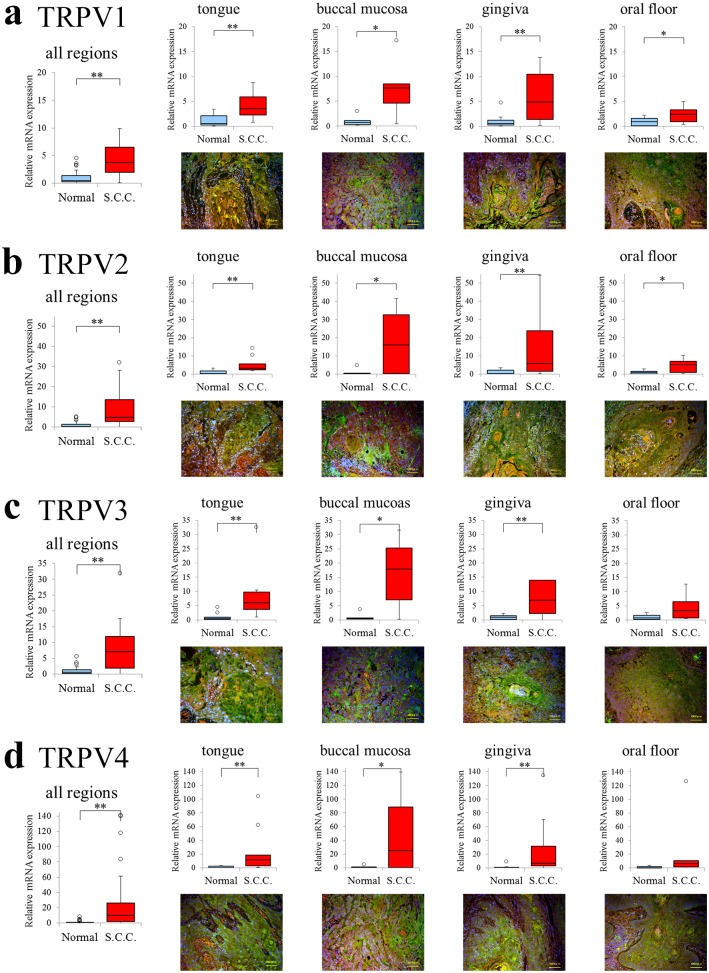
Comparisons of relative levels of expression of *TRPV1–4* in all oral cavity sites between normal oral mucosae and oral mucosae from patients with SCC. The levels of each target gene were normalized to those of *ACTB* in the same samples and are expressed relative to expression in the control group (DDCT method; Applied Biosystems). Box plots illustrate the median, lower, and upper quartiles of *TRPV1–4* expression. Whiskers were calculated using the Tukey method; filled circles represent outliers. **p < 0.01, *p < 0.05. Immunostaining images of squamous cell cancer in each respective site are presented below the graphs. Merged image of anti-TRPV1-4 (green), counterstained with Evance blue (red) and DAPI (blue). Comparisons of relative expression levels of **a.** *TRPV1*; **b.** *TRPV2*; **c.** *TRPV3*; and **d.** *TRPV4*.

Expression of *TRPV1–4* mRNA was confirmed and quantified in all oral SCC sites. Comparison between overall oral cavity mRNA expression in normal mucosae and mucosae from patients with SCC revealed that expression of all *TRPV*s was significantly higher in oral mucosae from patients with SCC (P < 0.01; Fig 4). Site-by-site comparisons demonstrated that *TRPV1–4* mRNA expression was significantly higher in oral mucosae from patients with SCC than in normal mucosae for almost all sites, with only the increased expression of *TRPV3* (Fig 4c) and *TRPV4* (Fig 4d) in the oral floor not determined to be statistically significantly (Fig 4).

#### Differences in mRNA expression by pathological grade of oral SCC

**Fig 5:**
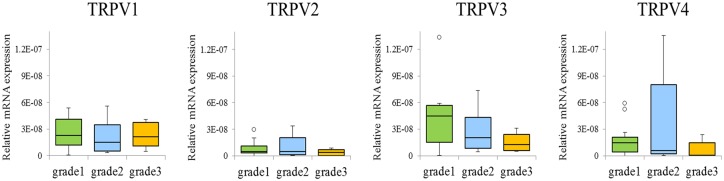
Box plot of relative levels of expression of *TRPV1*, *2*, *3*, and *4* compared by tumor grade. Expression levels of each target gene were normalized to those of *ACTB* in the same sample and are expressed relative to control group expression (DDCT method; Applied Biosystems). Box plots illustrate the median, lower, and upper quartiles of *TRPV1–4* expression. Whiskers were calculated using the Tukey method; filled circles represent outliers. No consistent trends or significant differences in relative levels of expression of *TRPV1*, *2*, *3*, or *4* were observed according to tumor grade of human oral SCC.

Given that neoplastic transformation resulted in increased *TRPV* mRNA expression, relative to normal mucosae, we predicted that there may be differences in *TRPV* expression relative to tumor grade; however, we did not observe any significant differences between tumor grades, nor did we observe any indications of tendencies towards differences between them (Fig 5).

### Effects of alcohol consumption and smoking on *TRPV1–4* expression in normal mucosae

**Fig 6:**
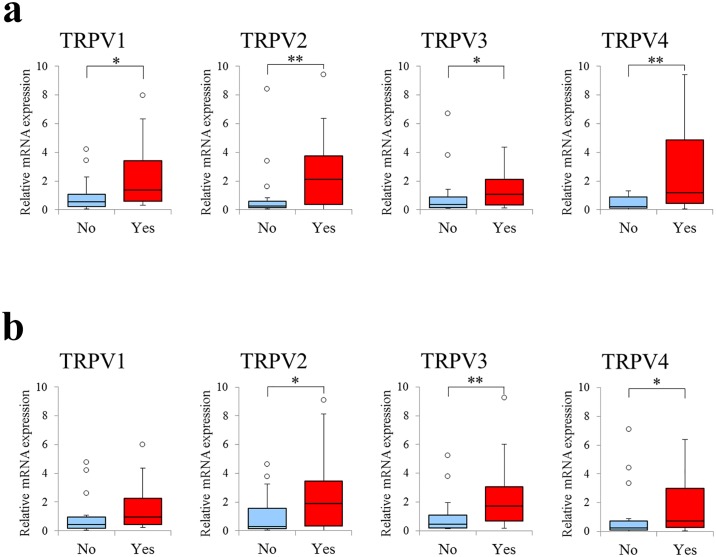
Box plot illustrating relative levels of *TRPV1*, *2*, *3*, and *4* expression compared by alcohol consumption and smoker status. Expression levels of each target gene were normalized to those of *ACTB* in the same samples and are expressed relative to control group expression levels (DDCT method; Applied Biosystems). Box plots illustrate the median, and lower, and upper quartiles of *TRPV1–4* expression. Whiskers were calculated using the Tukey method; filled circles represent outliers. **p < 0.01, *p < 0.05. (a) Relative expression levels of *TRPV1*, *2*, *3*, and *4* were all significantly higher in alcohol users than non-users. (b) Relative expression levels of *TRPV1*, *2*, *3*, and *4* were higher in the smoking than the non-smoking group. For *TRPV2*, *3* and *4*, the difference was significant, whereas for *TRPV1*, the difference was not significant, although expression levels of this receptor were higher among smokers.

Of the 37 patients, 20 had a history of alcohol consumption, while 18 had a history of smoking. Because alcohol consumption and smoking are risk factors for cancer, we hypothesized that external stimulus-induced changes in the oral environment in these patients would result in increased *TRPV* expression and greater sensitivity to external stimuli. We found that patients with a history of alcohol consumption demonstrated significantly higher relative mRNA expression levels of all *TRPV*s (Fig 6a). Patients with a history of smoking also demonstrated increased levels of expression of all TRPVs and this difference was significant for *TRPV2*, *TRPV3*, and *TRPV4* (Fig 6b). Insufficient numbers of patients in both the alcohol consumption and smoking groups meant that we were unable to conduct meaningful statistical comparisons of expression levels in the individual oral cavity sites.

## Discussion

TRPV family sensory receptors respond to external stimuli and are expressed in sensory neurons [1, 23]; however, recent studies have reported that these proteins are also expressed in non-neural cells, including the skin, intestine, lungs, and bladder [5, 6, 24, 25]. Although it is not yet fully understood how TRPV receptors convert external stimuli into intracellular signals, they can induce both cell apoptosis and, conversely, cell proliferation.

In the present study, we quantitatively and qualitatively confirmed the expression of TRPV1–4 in the gingiva, tongue, oral floor, and buccal mucosa of normal human oral cavities. Our results, indicating expression of all four receptors, suggest that heat and chemical substances, such as capsaicin, may have an effect on oral mucosae. Our data reveal relatively high expression levels of *TRPV1* and *3* compared to *TRPV2* and *4*, while expression of all *TRPV*s in normal oral mucosae was greater in the tongue and oral floor than the gingiva and buccal mucosa. We predicted that TRPV2 expression would be low, since it is only sensitive to temperatures ≥ 52°C, which are seldom encountered in daily life. However, TRPV4 expression was also relatively low, despite little overlap in the range of sensitivity of this receptor with that of TRPV2; thus, we surmise that the frequency of exposure to temperature stimuli is not correlated with the level of expression of TRPV receptors.

Since both the mechanism conversion of external stimuli to intracellular signals and the efficiency of this conversion are unknown, it is difficult to correlate TRPV expression levels with their function. However, we were able to determine a consistent histological expression pattern. Furthermore, we observed that levels of expression differed among sites in the oral cavity, indicating that stimuli signal conversion may differ according to the site in which they are experienced.

The TRPV family consists of transient receptor potential cation channels, which respond to external stimuli and which do not work through second messengers, but rather exhibit direct intermolecular interactions. It is highly likely that their actions affect cell behavior, given that the expression of TRPV proteins results in the formation of an ion gradient following stimulus reception. In epithelial cells of the epidermis and mucosae, signal transduction between adjacent cells occurs by ion gradient transduction via gap junctions. Together, this information leads us to speculate that TRPV-mediated external stimuli may be transmitted to adjacent cells, engendering local changes. In the future, it will be of interest to determine the mechanism of signal conversion to output via TRPV receptors in normal oral mucosae.

Comparisons of *TRPV* expression levels in normal human mucosae and oral mucosae from patients with SCC revealed that expression was higher in SCC cases at all sites. There have been various studies of TRPV expression in cancer cells, including rectal cancer, urinary tract cancers (bladder, prostate, etc.), lung cancer, and hepatoblastoma [7–10]. In many reports to date, the action of agonists on TRPV1 has been found to inhibit cell migration; therefore, capsaicin and related TRPV1 agonists are considered to have the potential to inhibit cancer progression, leading to anticipation that they may be effective as antitumor drugs. However, in cancers such as hepatoblastoma, TRPV1 signals activate and stimulate migration of vascular endothelial cells, thus creating an opposing expectation that TRPV1 antagonists may have anticancer effects [10]. Further careful studies are required to determine whether the effects of TRPV1 signaling differ according to tumor site. Furthermore, stimulation of TRPV4 promotes, not only tumor cell migration, but also neovascularization [26, 27], suggesting a possible relationship with hematogenous dissemination. However, it is not clear how stimuli would activate TRPV receptors in these organs. TRPV receptors are present; however, environmental factors known to activate them are absent from the immediate environments of such organs. In contrast, the oral environment is exposed to the type of stimuli that activate TRPV receptors, thus, it may provide an important model for understanding the relationship between TRPV receptors and cancer.

While the effects of TRPV signals in oral SCC require further investigation, epidemiological evidence indicating that external stimuli are risk factors for malignant tumor development supports the hypothesis that TRPV antagonists may have antitumor effects. However, the TRPV1 agonist, capsaicin, can inhibit proliferation of oral SCC. Gonzales et al found that capsaicin could induce apoptosis in isolated and cultured oral SCC cells, or when administered into tumor lesions by local injection [16]; they concluded that the mechanism of action was cell death due to the generation of reactive oxygen species independent of TRPV1-mediated signal transduction. Moreover, other sources of evidence both support and oppose a role for capsaicin as an oncogenic factor, since no epidemiological studies have found that people from cultures where capsaicin is habitually consumed display higher rates of oral cancer, whereas long-term use of capsaicin cream has been shown to be a risk factor for the development of skin cancer [28]. In addition, multiple pathways for carcinogenic and antitumor effects of capsaicin have been identified, indicating that capsaicin may demonstrate both effects depending on its concentration.

We found that smoking and alcohol consumption increase *TRPV* expression in normal oral mucosae. Smoking and alcohol consumption are risk factors for oral cancer [29–32] and increased levels and activity of TRPV may be involved in oncogenic transformation of normal cells. If the activation of TRPV in normal cells results in cell proliferation, then the external stimuli of tobacco and alcohol would be expected to trigger a negative cycle of neoplasia. No specific chemical substances in alcohol or tobacco smoke have been shown to act as TRPV agonists; thus, it seems unlikely that expression of these receptors would be upregulated because of TRPV signaling induced by alcohol or tobacco. Rather, it is more likely that the action of alcohol and tobacco on normal mucosa triggers cell transformation, thereby resulting in up-regulated receptor expression. It may be possible to demonstrate a correlation between TRPV expression levels and the level of risk of oncogenic transformation by examining TRPV expression in leukoplakia, a premalignant oral lesion. Thus, by measuring *TRPV* mRNA levels in samples scraped from the oral mucosae of habitual smokers and drinkers, it may be possible to demonstrate the level of risk of oncogenesis as an early warning/screening test. However, leukoplakia diagnosis requires detailed pathologic assessment of the resected lesion, and specimen collection is ethically problematic; thus, we were unable to conduct such an examination. Studies using animal models may be necessary in the future.

Although we did not observe any significant differences in expression of individual *TRPV* receptors in specific oral cavity sites, levels of all four were relatively high in the tongue and oral floor, compared with those in the buccal mucosa and gingiva. Oral cancer occurs frequently in the tongue and oral floor, and only rarely in the buccal mucosa and gingiva [31]. Hence, our data support the hypothesis that increased sensitivity to external stimuli, due to up-regulated expression of TRPV, promotes neoplastic transformation, although the function of TRPV receptors in oral mucosae and in oral SCC remains to be determined.

Activation of TRPV can induce the release of inflammatory cytokines [33], which can be recognized as pain by pain receptors. In fact, recognition of capsaicin exposure via TRPV1 is well known to be perceived as pain; however, to date, this phenomenon has only been demonstrated in the nervous system. Many cancer patients complain of intense localized pain, while our data shows up-regulated expression of TRPV in cancer tissue, suggesting that overexpression of TRPV is likely to be a factor in enhanced sensitivity to external stimuli.

In summary, the patterns of TRPV receptor expression in normal oral mucosae and oral mucosae from patients with SCC represent interesting findings. Further research is required to determine the relationship between external stimuli and neoplastic transformation. Expression of all *TRPV* genes assessed was greater in oral mucosae from patients with SCC than in normal oral mucosae, demonstrating that various external stimuli may affect the behavior of cancer cells. These findings are anticipated to contribute to the treatment and prevention of cancers caused by external stimuli.

## Supporting Information

S1 Appendix Immunostaining images of TRPV1–4 in human squamous cell carcinoma of the buccal mucosae (by grade). Merged image of anti-TRPV1–4 (green), counterstained with Evance blue (red) and DAPI (blue).
(TIF)

S2 Appendix Immunostaining images of TRPV1–4 in human squamous cell carcinoma of the gingiva (by grade). Merged image of anti-TRPV1–4 (green), counterstained with Evance blue (red) and DAPI (blue).
(TIF)

S3 Appendix Immunostaining images of TRPV1–4 in human squamous cell carcinoma of the oral floor (by grade). Merged image of anti-TRPV1–4 (green), counterstained with Evance blue (red) and DAPI (blue).
(TIF)
