# Supplementary figures and images for: Upregulated Expression of Transient Receptor Potential Cation Channel Subfamily V Receptors in Mucosae of Patients with Oral Squamous Cell Carcinoma and Patients with a History of Alcohol Consumption or Smoking
Source: PLoS One. 2017 Jan 12;12(1):e0169723. doi: 10.1371/journal.pone.0169723 (PMC5230781; doi:10.1371/journal.pone.0169723)

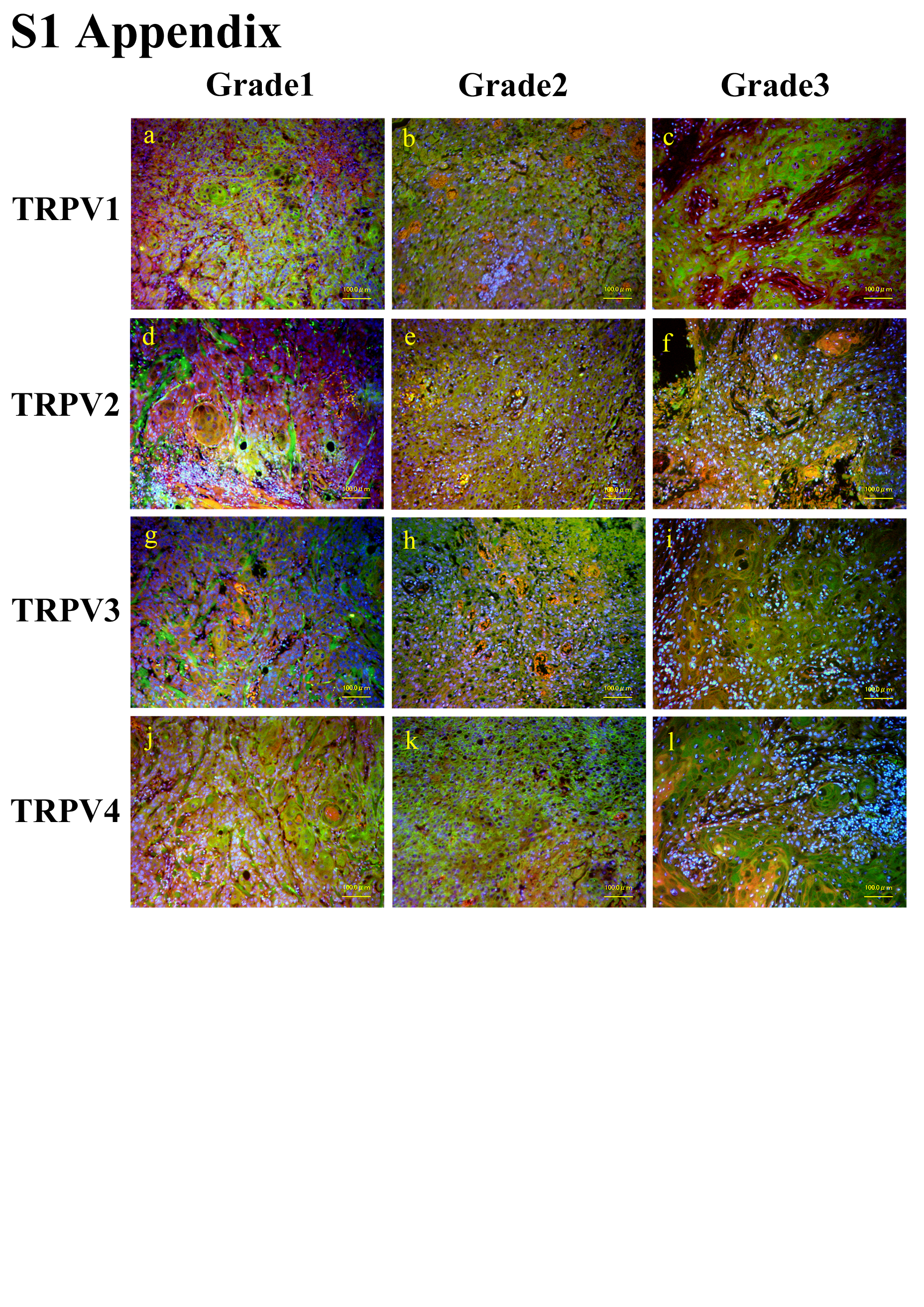

Supplement: S1 Appendix — Merged image of anti-TRPV1–4 (green), counterstained with Evance blue (red) and DAPI (blue). (TIF) [file pone.0169723.s001.tif]

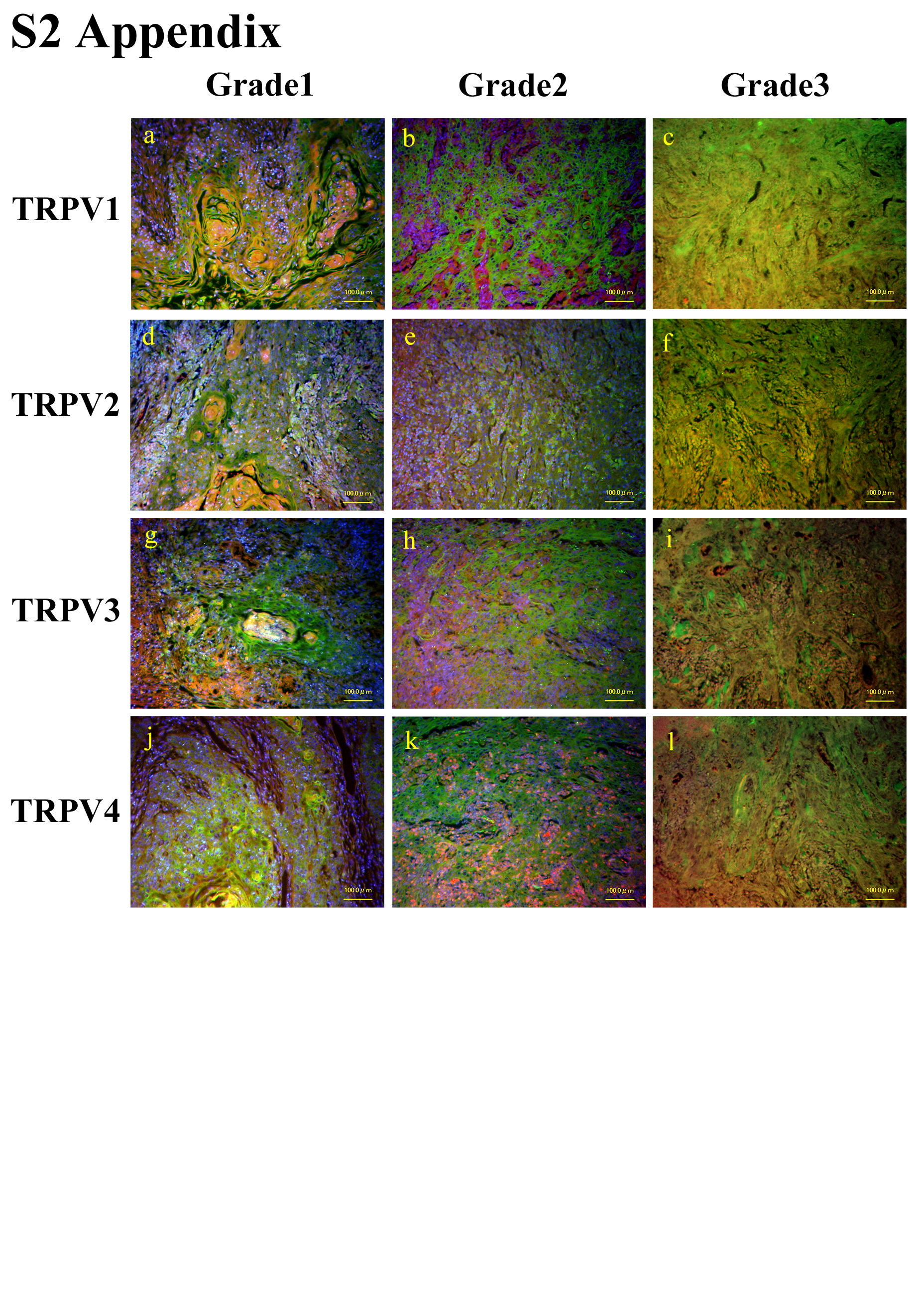

Supplement: S2 Appendix — Merged image of anti-TRPV1–4 (green), counterstained with Evance blue (red) and DAPI (blue). (TIF) [file pone.0169723.s002.tif]

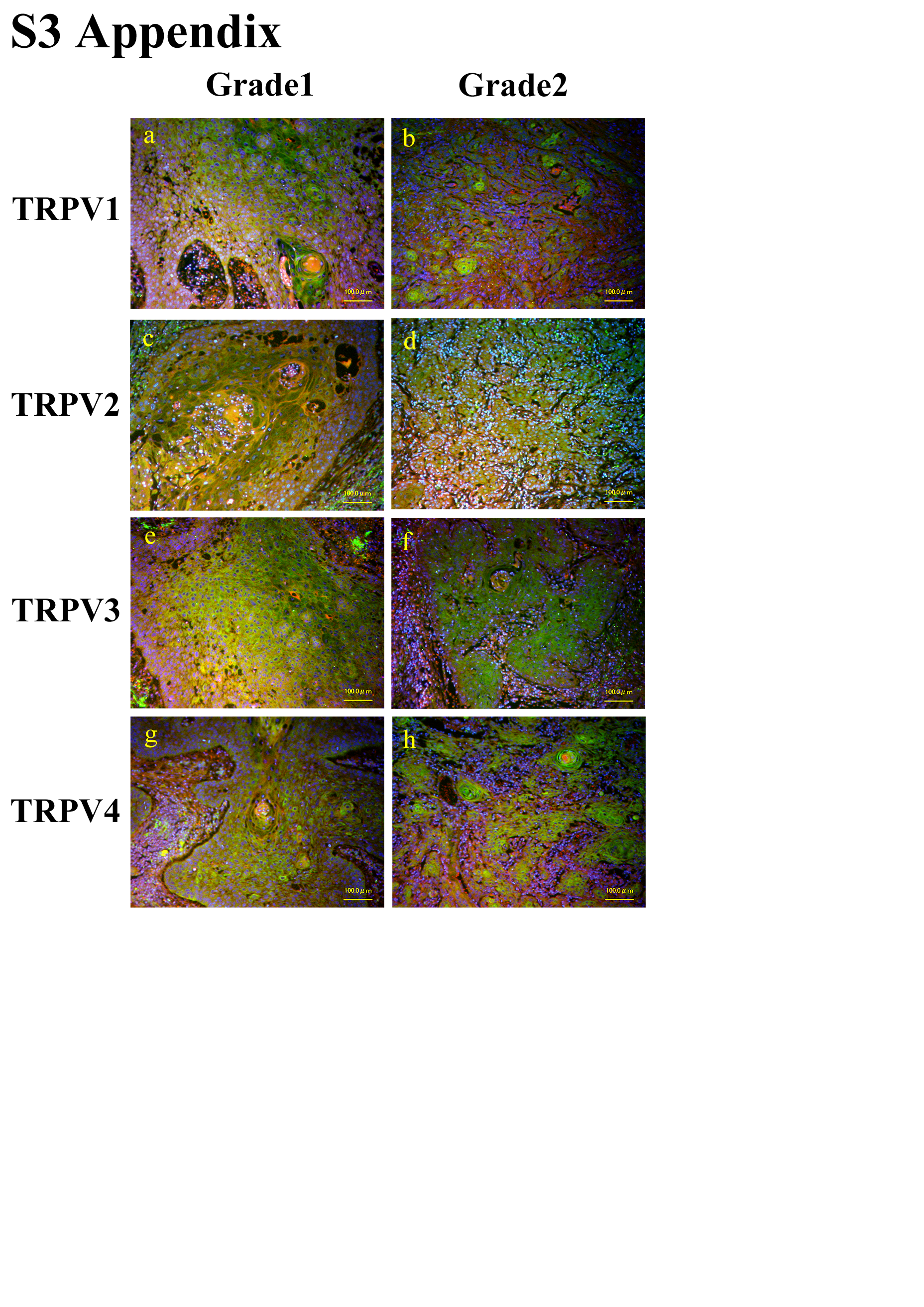

Supplement: S3 Appendix — Merged image of anti-TRPV1–4 (green), counterstained with Evance blue (red) and DAPI (blue). (TIF) [file pone.0169723.s003.tif]
